# Partial body fat percentage as a predictor of fragility fractures in a large cohort: a cross-sectional study

**DOI:** 10.1093/rap/rkae010

**Published:** 2024-01-23

**Authors:** Hamzah Amin, Fauzaan Ali Syed, Muhammed Aqib Khan, Zain Sultan, Marwan Bukhari

**Affiliations:** Lancaster Medical School, Faculty of Health and Medicine, Lancaster University, Lancaster, UK; Lancaster Medical School, Faculty of Health and Medicine, Lancaster University, Lancaster, UK; Department of Rheumatology, University Hospitals of Morecambe Bay NHS Foundation Trust, Morecambe Bay, UK; Department of Rheumatology, University Hospitals of Morecambe Bay NHS Foundation Trust, Morecambe Bay, UK; Lancaster Medical School, Faculty of Health and Medicine, Lancaster University, Lancaster, UK; Department of Rheumatology, University Hospitals of Morecambe Bay NHS Foundation Trust, Morecambe Bay, UK

**Keywords:** body fat percentage, fragility fractures, fracture risk estimation

## Abstract

**Objectives:**

BMI is a component of fracture risk calculators; however, it may be too simplistic to predict fracture risk. There is emerging evidence for the role that fat plays as a predictor of fracture. Partial body fat percentage (PBF%) may be a novel way to predict both hip and non-hip fractures. The aim of this study is to evaluate PBF% as a predictor of fragility fractures.

**Methods:**

A multivariate logistic regression analysis was conducted looking at PBF% as a predicter of both non-hip and hip fractures in an observational cohort. Our results were adjusted for age, biological sex, gender, smoking status, excess alcohol consumption (>3 units/day), current steroid therapy and the T-scores in both femurs. To allow for comparison, the same model was used with BMI, height and weight as the primary predictor of fracture. A subgroup analysis was conducted stratified by fracture site. A sensitivity analysis using a negative binomial regression was conducted.

**Results:**

A total of 31 447 patients were included in our analysis [mean age 64.9 years (s.d. 12.9)]. PBF% was shown to predict all non-hip fractures after adjustment [odds ratio (OR) 22.14 (95% CI 15.08, 32.50)]. Hip fractures were not predicted by our model [OR 4.19 (95% CI 0.43, 41.46)]. Sensitivity analysis demonstrated a lack of predictive capability for hip fracture but not non-hip fractures.

**Conclusion:**

PBF% may be a suitable predictor for all non-hip fractures, independent of confounding variables. More research is needed on whether it can predict hip fractures.

Key messages

- Partial body fat percentage is a good predictor of non-hip fractures.
- Partial body fat percentage did not predict hip fractures in our population.
- Fracture risk calculators may be underestimating the risk of non-hip fractures in the clinically overweight population.

## Introduction

Osteoporosis and fragility fractures represent a tremendous cost to societies and individuals worldwide [1]. With an ageing population, it is expected that the incidence of these fractures will increase [2]. Fracture risk estimation tools are used to identify ‘at-risk’ patients and help with referral for subsequent in-depth evaluation via dual-energy X-ray absorptiometry (DEXA). QFracture^®^ and FRAX^®^, both of which are fracture risk estimation tools, are commonly used in practice and incorporate height and weight into their algorithms. Height and weight are used to calculate the body mass index (BMI). BMI is very easy to obtain clinically, however, it may be too simplistic to calculate fracture risk.

There is an intricate relationship between height, weight, BMI and subsequent fracture risk. Increased height, decreased weight and decreasing BMI are all associated with fragility fractures, especially in elderly women [3–7]. Furthermore, at a population level, increases in BMI are thought to be protective of fracture and osteoporosis [5]. This protective effect is seen until a BMI of 40 [8]. Meta-analyses have demonstrated a non-linear relationship between fracture risk and BMI, with the majority of the effects being mediated by weight as opposed to height in non-hip fractures [9, 10]. Weight is thought to be protective of fracture, as heavier patients have increased loading through bones and subsequently have higher bone density. However, the composition of this weight (i.e. muscle mass *vs* fat mass) is also of importance in terms of fracture risk and is currently not considered in fracture risk calculators.

Adipose tissue plays an important role in bone loss by promoting a chronic low-grade inflammatory state [11], oxidative stress on tissues [12] and, finally, creating hormonal changes that are detrimental to bone [13]. Elderly people also undergo compositional changes in body fat, namely a decrease in subcutaneous fat and an increase in visceral fat, further adding to the catabolic effects of adipose tissue on bone [14]. Furthermore, muscle mass loss in the elderly is common and adds to fracture risk by reducing physical performance and stability [15]. However, muscle mass has shown not to be a good predictor of fracture [16]. Consequently, given the physiological effects of adiposity on bone, it may be better suited to predict fracture risk than the current approach of BMI.

The literature is undecided on the relationship between body fat and fragility fractures. Pana *et al.* [17] highlighted an association between increased total body fat percentage (TBF%) and the risk of hip fractures in males, but this was not echoed across non-hip sites. In females, increased TBF% was protective for hip fractures, but not other fragility fractures [17]. A cross-sectional study conducted by Hsu *et al.* [18] on a Chinese cohort suggested increased TBF% resulted in an increased risk of osteoporosis and its associated fragility fractures in both men and women. Other studies have highlighted no association between body fat and fracture risk [19]. Thus further research is needed to allow a greater understanding on the effect of body fat as a predictor of fracture.

While obtaining TBF% using a full-body DEXA scan is considered the gold standard, this is neither practical nor cost effective for all patients. However, patients often undergo a DEXA scan of the lumbar spine and hip regions, hence many patients will have a so-called partial body fat percentage (PBF%) value, which may be a new novel predictor of fracture risk.

The aim of this study was to investigate the relationship between PBF% and fracture risk. Specifically, whether PBF% is able to predict both hip and non-hip (also referred to as major fragility fracture) fractures. We also aimed to assess BMI, height and weight as known predictors of fracture and contrast this with PBF% as a novel predictor of fracture.

## Methods

Patients were referred to a regional DEXA scanner in the northwest of England between 2004 and 2019 from either primary or secondary care. At each scan, patients were given a questionnaire to assess for certain risk factors such as smoking and alcohol intake. This was correlated to the patients’ medical history, including previous fractures. Height and weight data were collected by the technician at each appointment. Our patients underwent DEXA scanning at the L1–L4 regions as well as at both hips. We also had access to the lean and fat masses at all of these regions. To work out the PBF% we used the following equation: (abdominal fat mass + left hip fat mass + right hip fat mass)/(total fat mass at these sites + total lean mass at these sites) × 100.

Statistical analysis was performed using Stata version 12 (StataCorp©, College Station, TX, USA). A logistic model reporting the odds ratio (OR) was used. The model was fitted adjusting for known predictors of fractures including age, gender, current smoking status, current excess alcohol intake (>3 units/day), current steroid therapy and the T-scores in the left and right femurs. We decided to use both the left and right hip T-scores, as previous studies have highlighted underestimation of osteoporosis when using only a single measurement [20].

We first analysed PBF% independently to get an unadjusted baseline effect. Subsequently a stepwise approach was used. This model was then applied to both non-hip and hip fractures. The same model was used for height, weight and BMI separately for comparison. A subgroup analysis was conducted on particular anatomical sites of fractures, adjusting for all the above variables. We also conducted a sensitivity analysis utilising a negative binomial regression reporting incidence rate ratios (IRRs).

Full ethical approval for the use and extraction of pseudo-anonymised data was given by the local ethics committee [National Research Ethics Service (NRES) committee Northwest Preston (project number 14/NW/1136)].

## Results

A total of 31 447 patients were included in our analysis: 5367 males and 26 080 females. The mean age at the time of the scan was 64.9 years (s.d. 12.9). A total of 11 875 fractures were reported: 10 510 non-hip fractures (1641 fractures in males and 10 022 fractures in females) and 1365 hip fractures (311 in males and 1054 in females). The mean BMI was 27.1 (s.d. 7.5), height 162.0 cm (s.d. 8.6), weight 71.1 kg (s.d. 16.0) and PBF% 29.8% (s.d. 7.5). Table 1 shows these results stratified by gender.

**Table 1.** Population characteristics

Characteristics	Male	Female
Patients referred, *n* (%)	5390 (17.1)	26 156 (82.9)
Height, cm, mean (s.d.)	172.4 (7.6)	159.9 (7.1)
Weight, kg, mean (s.d.)	81.0 (15.9)	69.1 (15.2)
BMI, mean (s.d.)	27.2 (5.2)	27.1 (7.9)
PBF%, %, mean (s.d.)	26.7 (7.4)	30.5 (7.4)
Smokers, %	14.3	12.9
Patients consuming excess alcohol (>3 units/day), %	9.8	4.7
Patients on steroids, %	22.8	9.1
Patients reporting a non-hip fracture, %	30.5	36.0
Patients reporting a hip fracture, %	5.77	4.03

Unadjusted PBF% was predictive of fractures [OR 9.49 (95% CI 15.08, 32.50)]. This value decreased after adjustment for age, gender, smoking and alcohol status. However, when further adjusted for left and right femoral T-scores, this value increased. All results were statistically significant with *P* = 0.000. These results are shown in Table 2.

**Table 2.** Unadjusted and adjusted ORs for PBF% as a predictor for non-hip fracture

Variable	OR	95% CI
Unadjusted	9.18	6.98, 13.80
Adjusted for		
Age, biological sex	2.95	2.06, 4.24
Age, biological sex, current smoker, current alcohol excess, current steroid therapy	3.97	2.75, 5.72
Age, biological sex, current smoker, current alcohol excess, current steroid therapy, left femoral T-score, right femoral T-score	15.23	10.32, 22.48

In subsequent analyses, height, weight and BMI were inputted as the primary predictive variables, with all other confounding variables remaining the same. Only weight and PBF% were found to be predictive of fracture, with PBF% having higher odds of fracture compared with weight. Height and BMI were not related to fracture risk in our dataset Table 3.

**Table 3.** Fully adjusted ORs of height, weight, BMI and PBF% as predictors of non-hip fragility fractures

Variable	OR	95% CI
Height	1.00	1.00, 1.01
Weight	1.02	1.01, 1.02
BMI	1.00	1.00, 1.01
PBF%	15.23	10.32, 22.48

Next, using the same methods as above, we looked at hip fractures independently. The results of this analysis are shown in Table 4. Interestingly our results demonstrated that unadjusted PBF% was not associated with hip fractures. The majority of variables when adjusted into the analysis showed insignificance. After adjustment with the left and right femoral T-scores, the result was still statistically insignificant.

In our dataset, PBF% was not predictive of hip fracture. Height, weight and BMI were also not associated with hip fracture. The results of this analysis are shown in Table 5.

**Table 4.** Unadjusted and adjusted ORs for PBF% as a predictor for hip fractures

Variable	OR	95% CI
Unadjusted	3.26	0.54, 19.56
Adjusted for		
Age, biological sex	0.63	0.09, 4.20
Age, biological sex, current smoker, current alcohol excess, current steroid therapy	0.66	0.10, 4.45
Age, biological sex, current smoker, current alcohol excess, current steroid therapy, left femoral T-score, right femoral T-score	4.19	0.43, 41.46

**Table 5.** Fully adjusted ORs of height, weight, BMI and PBF% as predictors of hip fractures

Variable	OR	95% CI
Height	1.00	0.97, 1.02
Weight	1.01	1.00, 1.02
BMI	1.00	0.99, 1.01
PBF%	4.19	0.43, 41.46

Subgroup analysis demonstrated that following full adjustment, PBF% was able to predict fracture at all non-hip sites. PBF% did not predict hip fracture. The results of this analysis are shown in Table 6.

**Table 6.** Subgroup analysis of PBF% as a predictor of fragility fracture at specific anatomical locations

Location	OR	95% CI
Tibia/fibula	58.57	31.46, 109.07
Hip	4.19	0.42, 41.46
Forearm	3.66	2.27, 5.92
Humerus	17.55	6.92, 44.54
Spine	11.92	6.15, 23.08

Our sensitivity analysis further reinforced that PBF% was associated with non-hip fracture [IRR 5.11 (95% CI 3.81, 6.88), *P* = 0.000]. PBF% was not associated with hip fracture in our sensitivity analysis [IRR 4.13 (95% CI 0.42, 40.36), *P* = 0.222].

## Discussion

We found that PBF% was a good predictor of non-hip fractures, with specificity to all non-hip sites of fracture we analysed. The association between PBF% and major fragility fracture was strong. The subsequent sensitivity analysis confirmed the relationship between PBF% and non-hip fractures. However, one criticism that may arise from our results is the relatively large CIs seen in our subgroup analysis for certain anatomical sites of fracture, including the tibia, fibula and humerus. This is largely explained by the relatively small numbers of certain types of osteoporotic fragility fractures (spine, tibia, fibula and humerus) as well as the relative complexity of our model. Hence our data supports an approach of using PBF% as a predictor of all non-hip fractures rather than fractures at specific anatomical locations.

Our results challenges previous literature stating that body fat predicted fractures in women only [10, 17]. Our results also agree with the findings of Hsu *et al.* [18] that the risk of fracture is higher in patients with increased body fat percentage. We also agree with the literature highlighting that the biggest risk factor for non-hip fragility fracture in terms of body composition is patient weight, as can be seen in Table 3 [9, 10]. PBF% in our dataset seems to be a superior predictor of non-hip fragility fracture, which performs better than the current approach of using BMI in our population. PBF% may be a new pragmatic way of predicting non-hip fracture in updated versions of fracture risk calculators.

PBF% was not associated with hip fractures in our population, with subsequent sensitivity analysis confirming this finding. We would have expected BMI and subsequently PBF% to be protective for hip fracture given that our patients were clinically overweight; however, this was not observed [17, 21]. While we did not see an association, larger prospective cohorts may still find an association between PBF% and hip fracture risk.

To our knowledge there are very limited studies looking at TBF% as a predictor of fracture. Some studies that have found an association between hip fractures and body fat have estimated TBF% and bone mineral density (BMD) using non-gold-standard approaches such as bioelectrical impedance (BEI) for fat mass and broadband ultrasound attenuation (BUA) for BMD [10, 17]. At best, these approaches only moderately correlate with the actual TBF% and BMD [22–24]. Hence the conclusions we can make from these studies are limited. Hsu *et al*. [18] used TBF% via a full-body DEXA scan; however, their analysis was conducted by looking at non-spine fractures collectively rather than hip and non-hip fractures. Hence it is difficult to extrapolate their findings to hip fractures specifically. Consequently, the true association between hip fracture and TBF% is yet to be determined. Hence it is difficult to compare our novel approach to previous studies looking at TBF%. Ultimately, further research using large prospective cohorts is needed to understand the relationship between TBF% and fracture risk. The correlation between TBF% and PBF% as predictors of fracture risk would then be useful in allowing us to understand the clinical utility of these approaches in fracture risk estimation.

While we looked at PBF%, this may not be the optimal approach to estimating hip fracture risk. A key reason for this could be explained by the importance of regional variation in adipose tissue (principally abdominal fat and gluteal fat), which may be being missed by using PBF%, TBF% and BEI. Sadeghi *et al.* [25], in their systematic review looking at abdominal obesity and the risk of hip fracture, found that abdominal obesity was associated with an increased risk of hip fracture. This association was found by analysing 295, 674 male and female patients who had prospective follow-up [25]. While all patients did not have abdominal obesity measured by DEXA, various measures were summed together in their review, including waist:hip ratio and waist circumference. An association was still found in their review. Furthermore, research investigating gynoid obesity demonstrated that patients with gynoid obesity had an increased propensity to fall [26]. Hence both abdominal and hip sites may play an important role in predicting fracture risk. While abdominal obesity [25] and fracture risk has been investigated, to our knowledge hip fat mass has not been researched as a predictor of fracture. Thus further research is needed to unpack the relationship between abdominal and hip fat as predictors of both non-hip and hip fractures. Research would also need to compare the predictive capability of total *vs* regional body fat approaches for fracture risk estimation.

## Limitations

Our study is limited by its design. We were only able to ascertain body fat percentage and fractures at a moment in time with no prospective follow-up. Another thing to consider is that fractures may lead to immobility, contributing to adiposity and muscle mass loss in this population—an unaccounted-for confounder. We also did not conduct analysis stratified by quintiles of PBF%, which may have revealed different findings. Some statistical uncertainty was observed in our results as indicated by some large CIs, however, this is due to the relatively small number of fractures we observed. We also recognize that our population of referred patients may not represent the general population, as our study consisted of 99% Caucasian people, hence a cohort that matches the general population would be needed to further interrogate our findings.

However, our study has strengths in that we have analysed >30 000 patients, with a large number being male patients compared with previous studies. Also, the magnitude of the ORs seen for PBF% and non-hip fractures indicate a likely direct causal relationship between the two. Our study is also strengthened by the use of the gold-standard approach of using DEXA for both the measurement of BMD and PBF%.

## Conclusions

PBF% was able to predict non-hip fractures, with a sensitivity analysis confirming the relationship. PBF% did not predict hip fracture in our dataset. PBF% may be a viable predictor of non-hip fracture, but its use in hip fracture estimation is yet to be determined. We recommend that further research is needed to establish a relationship between TBF%, PBF% and hip fractures. Further research investigating how regional variation in adiposity predicts fracture risk may allow us to better predict hip fractures.

## Data Availability

Data can be made available upon reasonable request to the corresponding author.

## References

[1] Clynes MA , Harvey NC, Curtis EM et al The epidemiology of osteoporosis. Br Med Bull 2020;133:105–17. 32282039 10.1093/bmb/ldaa005 PMC7115830

[2] Hernlund E , Svedbom A, Ivergård M et al Osteoporosis in the European Union: medical management, epidemiology and economic burden. A report prepared in collaboration with the International Osteoporosis Foundation (IOF) and the European Federation of Pharmaceutical Industry Associations (EFPIA). Arch Osteoporos 2013;8:136. 24113837 10.1007/s11657-013-0136-1 PMC3880487

[3] Cummings SR , Nevitt MC, Browner WS et al Risk factors for hip fracture in white women. N Engl J Med 1995;332:767–73. 7862179 10.1056/NEJM199503233321202

[4] Ensrud KE , Lipschutz RC, Cauley JA et al Body size and hip fracture risk in older women: a prospective study. Am J Med 1997;103:274–80. 9382119 10.1016/s0002-9343(97)00025-9

[5] Johansson H , Kanis JA, Odén A et al A meta-analysis of the association of fracture risk and body mass index in women. J Bone Miner Res 2014;29:223–33. 23775829 10.1002/jbmr.2017

[6] Armstrong ME , Kirichek O, Cairns BJ et al Relationship of height to site-specific fracture risk in postmenopausal women. J Bone Miner Res 2016;31:725–31. 26572496 10.1002/jbmr.2742 PMC4832288

[7] Honkanen RJ , Honkanen K, Kröger H et al Risk factors for perimenopausal distal forearm fracture. Osteoporosis Int 2000;11:265–70. 10.1007/s001980050291 10824244

[8] Oldroyd A , Mitchell K, Bukhari M. The prevalence of osteoporosis in an older population with very high body mass index: evidence for an association. Int J Clin Pract 2014;68:771–4. 24447370 10.1111/ijcp.12371

[9] De Laet C , Kanis JA, Odén A et al Body mass index as a predictor of fracture risk: a meta-analysis. Osteoporos Int 2005;16:1330–8. 15928804 10.1007/s00198-005-1863-y

[10] Moayyeri A , Luben RN, Wareham NJ, Khaw KT. Body fat mass is a predictor of risk of osteoporotic fractures in women but not in men: a prospective population study. J Intern Med 2012;271:472–80. 21848670 10.1111/j.1365-2796.2011.02443.x

[11] Lumeng CN , Saltiel AR. Inflammatory links between obesity and metabolic disease. J Clin Invest 2011;121:2111–7. 21633179 10.1172/JCI57132 PMC3104776

[12] Zhang C , Li H, Li J et al Oxidative stress: a common pathological state in a high-risk population for osteoporosis. Biomed Pharmacother 2023;163:114834. 37163779 10.1016/j.biopha.2023.114834

[13] Savvidis C , Tournis S, Dede AD. Obesity and bone metabolism. Hormones (Athens) 2018;17:205–17. 29858847 10.1007/s42000-018-0018-4

[14] Ponti F , Santoro A, Mercatelli D et al Aging and imaging assessment of body composition: from fat to facts. Front Endocrinol 2019;10:861. 10.3389/fendo.2019.00861 PMC6970947 31993018

[15] Alajlouni D , Bliuc D, Tran T et al Decline in muscle strength and performance predicts fracture risk in elderly women and men. J Clin Endocrinol Metab 2020;105:dgaa414. 32639571 10.1210/clinem/dgaa414

[16] Alajlouni DA , Bliuc D, Tran TS, Blank RD, Center JR. Muscle strength and physical performance contribute to and improve fracture risk prediction in older people: a narrative review. Bone 2023;172:116755. 37028582 10.1016/j.bone.2023.116755

[17] Pana TA , Kioh SH, Neal SR et al Body fat percentage and the long-term risk of fractures. The EPIC-Norfolk prospective population cohort study. Maturitas 2023;168:71–7. 36502648 10.1016/j.maturitas.2022.11.005 PMC7614563

[18] Hsu YH , Venners SA, Terwedow HA et al Relation of body composition, fat mass, and serum lipids to osteoporotic fractures and bone mineral density in Chinese men and women. Am J Clin Nutr 2006;83:146–54. 16400063 10.1093/ajcn/83.1.146

[19] Hong C , Choi S, Park M, Park SM, Lee G. Body composition and osteoporotic fracture using anthropometric prediction equations to assess muscle and fat masses. J Cachexia Sarcopenia Muscle 2021;12:2247–58. 34706399 10.1002/jcsm.12850 PMC8718033

[20] Hwang HJ , Park SY, Lee SH, Han SB, Ro KH. Differences in bone mineral density between the right and left hips in postmenopausal women. J Korean Med Sci 2012;27:686–90. 22690102 10.3346/jkms.2012.27.6.686 PMC3369457

[21] Tang X , Liu G, Kang J et al Obesity and risk of hip fracture in adults: a meta-analysis of prospective cohort studies. PLoS One 2013;8:e55077. 23593112 10.1371/journal.pone.0055077 PMC3625172

[22] Nayak S , Olkin I, Liu H et al Meta-analysis: accuracy of quantitative ultrasound for identifying patients with osteoporosis. Ann Intern Med 2006;144:832–41. 16754925 10.7326/0003-4819-144-11-200606060-00009

[23] Young H , Howey S, Purdie DW. Broadband ultrasound attenuation compared with dual-energy X-ray absorptiometry in screening for postmenopausal low bone density. Osteoporos Int 1993;3:160–4. 8481593 10.1007/BF01623278

[24] Nguyen HG , Lieu KB, Ho-Le TP, Ho-Pham LT, Nguyen TV. Discordance between quantitative ultrasound and dual-energy X-ray absorptiometry in bone mineral density: the Vietnam Osteoporosis Study. Osteoporos Sarcopenia 2021;7:6–10. 33869799 10.1016/j.afos.2021.03.003 PMC8044595

[25] Sadeghi O , Saneei P, Nasiri M, Larijani B, Esmaillzadeh A. Abdominal obesity and risk of hip fracture: a systematic review and meta-analysis of prospective studies. Adv Nutr 2017;8:728–38. 28916573 10.3945/an.117.015545 PMC5593104

[26] Neri SGR , Tiedemann A, Gadelha AB, Lima RM. Body fat distribution in obesity and the association with falls: a cohort study of Brazilian women aged 60 years and over. Maturitas 2020;139:64–8. 32747043 10.1016/j.maturitas.2020.06.009

